# Comparative Physical and Mechanical Characterization of Lightweight Porous Materials: Bulk Density as Indicator of Mechanical Properties

**DOI:** 10.3390/ma19204346

**Published:** 2026-10-15

**Authors:** Malwina Kolano

**Affiliations:** Faculty of Civil Engineering and Resource Management, AGH University of Krakow, 30-059 Krakow, Poland; mkolano@agh.edu.pl

**Keywords:** lightweight porous materials, autoclaved aerated concrete, foamed concrete, rigid polyurethane foam, bulk density, mechanical properties

## Abstract

Lightweight porous materials are widely used in construction due to their low density and favorable thermal insulation properties; however, their mechanical performance depends on factors extending beyond bulk density alone. The aim of this study was to perform a comprehensive physical and mechanical characterization of three representative lightweight porous materials belonging to different material classes: autoclaved aerated concrete (AAC), foamed concrete, and rigid polyurethane foam, within a common experimental and analytical framework. The investigation included the determination of particle density, bulk density, porosity, unconfined compressive strength (UCS), Young’s modulus, and shear strength parameters obtained from triaxial compression tests. In addition, correlations between bulk density and selected mechanical properties were analyzed for each material. The mean bulk density, porosity, and UCS values were 566 kg/m^3^, 73%, and 5.24 MPa for AAC; 518 kg/m^3^, 77%, and 0.88 MPa for foamed concrete; and 82 kg/m^3^, 93%, and 0.52 MPa for rigid polyurethane foam, respectively. Despite similar bulk densities, AAC and foamed concrete differed by approximately sixfold in UCS, eightfold in Young’s modulus, and sixfold in cohesion. Correlation analyses further showed that the density–property relationships were material-specific. The findings indicate that bulk density alone is not a universal predictor of the mechanical behavior of different classes of lightweight porous materials. A comprehensive characterization combining physical and mechanical properties is therefore required for reliable material evaluation and comparison.

## 1. Introduction

Lightweight porous materials are playing an increasingly important role in modern construction owing to their favorable strength-to-weight ratio, low thermal conductivity, and ability to reduce the self-weight of structures [1,2,3,4]. Their application contributes to improved building energy efficiency, reduced loads transferred to structural elements, and easier transportation and installation. Among the most commonly used lightweight porous materials are autoclaved aerated concrete (AAC), foamed concrete, and rigid polyurethane (PUR) foams, which are widely used as both structural and insulating materials [5,6,7,8,9]. Although all these materials are characterized by a high pore volume and low bulk density, they differ substantially in terms of manufacturing technology, pore formation mechanism, and the properties of the solid skeleton, which directly influence their mechanical behavior under loading.

Reducing material density generally improves thermal insulation performance; however, it also leads to a reduction in compressive strength, stiffness, and load-bearing capacity [5,10,11,12]. According to the theory of cellular solids proposed by Gibson and Ashby [13], the mechanical properties of porous materials depend not only on their density but also on the geometry of the pore structure and the properties of the material forming the solid skeleton. Numerous experimental studies have confirmed that the mechanical behavior of lightweight porous materials is governed not only by bulk density but also by porosity, pore size distribution, pore connectivity, the thickness of the solid walls, and the properties of the solid phase [4,9,14,15,16,17,18,19]. In rigid polyurethane foams, compressive strength and elastic modulus have also been shown to depend not only on density but also on the geometry and morphology of the cellular structure [20]. Consequently, materials with similar densities may exhibit substantially different mechanical properties due to differences in microstructure and pore formation mechanisms [7,14,15,16,17]. Therefore, although bulk density remains one of the fundamental parameters used to characterize lightweight porous materials, it should not be regarded as a universal predictor of their mechanical properties.

In recent years, numerous review papers on lightweight construction materials have emphasized the need for a comprehensive characterization that includes both physical and mechanical properties [4,11,15,17,18,19]. These studies recommend combining measurements of density, porosity, and microstructural parameters with strength and deformation tests to enable a reliable interpretation of the relationships between material structure and performance [4,8,17,18,19]. Such an approach provides a more comprehensive assessment than the analysis of individual physical or mechanical parameters alone. It has also been emphasized that a reliable evaluation of porous materials requires the simultaneous consideration of their microstructure, density, and mechanical response, as only their combined analysis enables a proper understanding of the relationships between material structure and behavior under loading [4,13,17].

Despite the rapidly increasing number of publications on lightweight construction materials, most studies focus on individual material groups, such as autoclaved aerated concrete, foamed concrete, lightweight aggregate concrete, cementitious composites, or rigid polyurethane foams [5,6,7,8]. The influence of mixture composition, lightweight aggregate type, mineral additives, foaming agents, modifications of the cellular structure, or reinforcement is typically investigated within a single material group, such as autoclaved aerated concrete, foamed concrete, or lightweight cementitious composites [4,5,7,8]. In contrast, studies enabling the direct comparison of different classes of lightweight porous materials within a common experimental and analytical framework that includes both physical and mechanical characterization remain scarce. At the same time, recent review papers have highlighted the need for standardized testing procedures that allow reliable comparisons between materials differing in composition, pore formation mechanism, and internal structure [4,15,17]. This issue is of considerable practical importance because lightweight porous materials are often classified according to their bulk density, which may incorrectly suggest similar mechanical properties for materials belonging to different classes despite substantial differences in their microstructure and manufacturing technology. As a result, there is still a lack of data allowing an unambiguous assessment of whether materials with similar bulk densities also exhibit comparable mechanical properties.

The aim of this study was to provide a comprehensive physical and mechanical characterization of three representative lightweight porous materials: autoclaved aerated concrete, foamed concrete, and rigid polyurethane foam. The investigation included the determination of particle density, bulk density, porosity, unconfined compressive strength, Young’s modulus, and shear strength parameters obtained from triaxial compression tests. The scientific contribution of the study lies in the direct cross-class comparison of materials with fundamentally different matrices and pore-formation mechanisms within a common experimental and analytical framework. Rather than examining density–property relationships within a single material class, the present study investigates whether relationships observed for one class of lightweight porous material can be transferred to another. The comparison is not intended to imply equivalence or interchangeability between the investigated material classes, but to evaluate the applicability of bulk density as a comparative indicator across materials with fundamentally different solid skeletons and pore-forming mechanisms. Particular attention was therefore given to materials with comparable bulk densities but different internal structures and manufacturing technologies. This approach enables the magnitude of cross-material differences in unconfined compressive strength, Young’s modulus, and shear strength parameters to be quantified and provides a basis for assessing the limitations of bulk density as a comparative parameter across different classes of lightweight porous materials.

## 2. Materials and Methods

### 2.1. Materials

Three lightweight porous materials with different material matrices were selected for the study: autoclaved aerated concrete (AAC), foamed concrete, and polyurethane foam. These materials represent different classes of engineering materials; however, they are all characterized by a well-developed porous structure and are widely used in construction and civil engineering.

#### 2.1.1. Autoclaved Aerated Concrete (Ytong)

Commercially available autoclaved aerated concrete (AAC) manufactured by Ytong (Xella Polska Sp. z o.o., Warsaw, Poland) was used in this study. As the manufacturer did not provide information regarding the material class, it was experimentally characterized based on its bulk density and mechanical properties.

**Figure 1:**
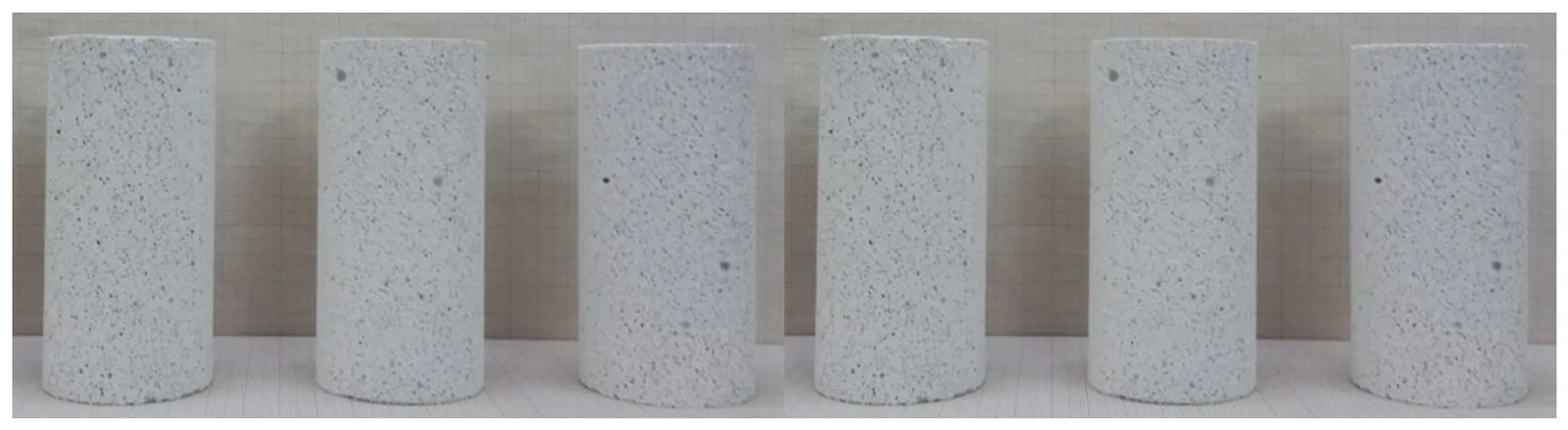
Representative specimens of autoclaved aerated concrete (Ytong) used in the study.

Twenty cylindrical specimens with a diameter of 50 mm and a height of 100 mm (height-to-diameter ratio of approximately 2:1) were prepared by cutting them from a block measuring 24 cm × 20 cm × 60 cm (Figure 1). The end surfaces of the specimens were prepared to ensure their mutual parallelism.

#### 2.1.2. Durafoam Foamed Concrete (Minova, Siemianowice Śląskie, Poland)

**Figure 2:**
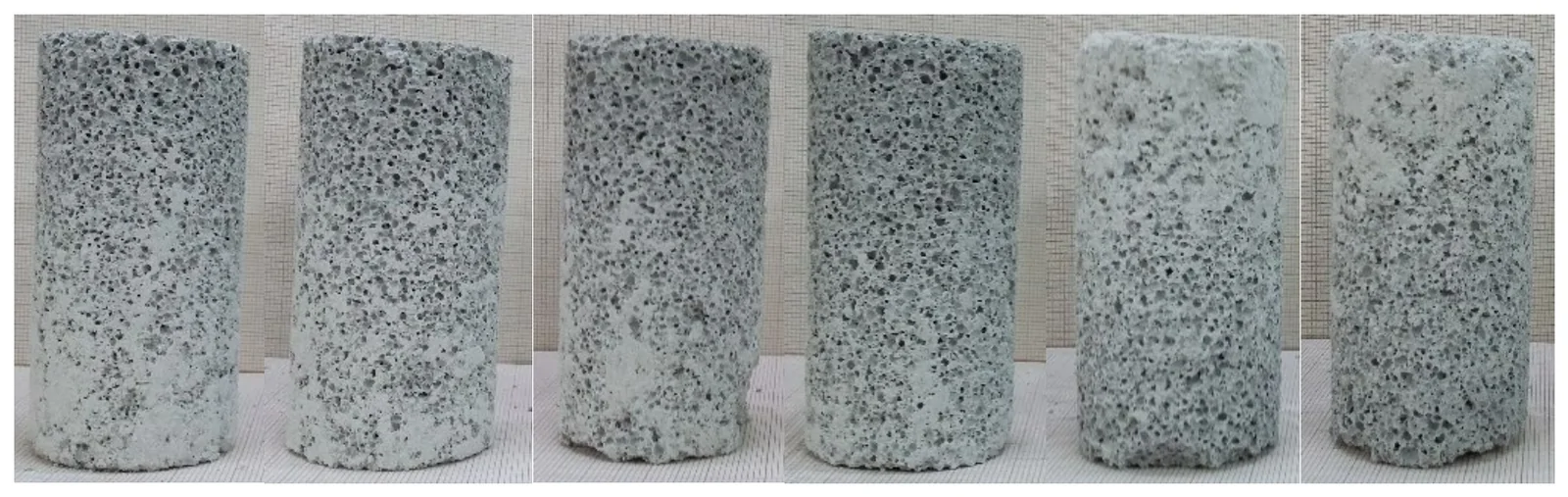
Representative specimens of Durafoam foamed concrete (Minova) used in the study.

The second material investigated was Durafoam foamed concrete supplied by Minova. Blocks measuring 1.0 m × 1.0 m × 0.3 m were used to prepare 30 cylindrical specimens with a diameter of 50 mm and a height of 100 mm (Figure 2). The end surfaces of the specimens were machined to ensure their mutual parallelism.

#### 2.1.3. Ekopur U7020 Polyurethane Foam (Minova, Siemianowice Śląskie, Poland)

The third material investigated was Ekopur U7020 rigid polyurethane foam (Minova), intended for the production of structural and insulating elements. It is a two-component polyurethane system consisting of component A (a mixture of polyols, additives, and a blowing agent) and component B (polymeric methylene diphenyl diisocyanate).

The foam was prepared in the Laboratory of the Department of Geomechanics, Civil Engineering and Geotechnics at AGH University of Krakow by manually mixing the two components using a high-speed mechanical mixer in accordance with the manufacturer’s recommendations.

**Figure 3:**
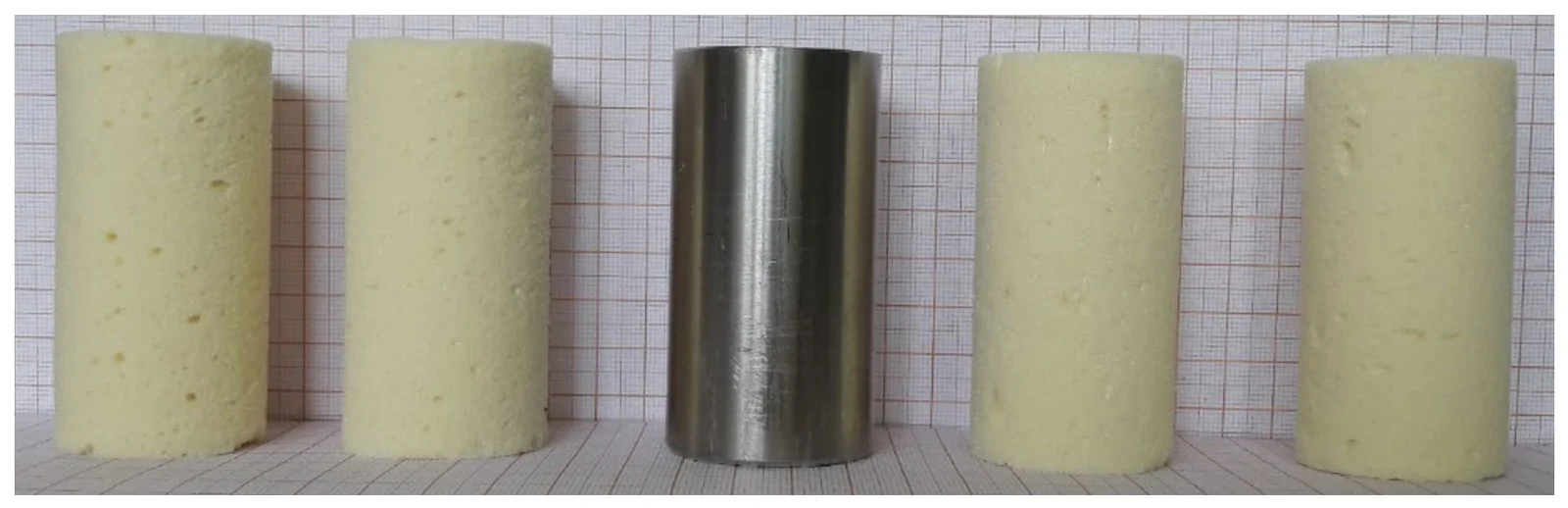
Representative specimens of Ekopur U 7020 polyurethane foam (Minova) used in the study.

After completion of the foaming process, 35 cylindrical specimens with a diameter of approximately 38 mm and a height of approximately 76 mm were cut from the prepared blocks for laboratory testing (Figure 3).

### 2.2. Methods

All laboratory tests were carried out in the Laboratory of the Department of Geomechanics, Civil Engineering and Geotechnics at AGH University of Krakow. The experimental program included the determination of physical properties (particle density, bulk density, and porosity), unconfined compressive strength (UCS), and shear strength parameters (cohesion, *c*, and internal friction angle, φ) obtained from triaxial compression tests. The PN-EN ISO 17892 [21,22,23,24] series was adopted as a common geomechanical testing framework for comparative characterization of the investigated porous materials. Although AAC, foamed concrete, and rigid polyurethane foam fall outside the primary scope of these geotechnical standards, the adopted procedures provide consistent loading conditions and parameter-evaluation principles for comparative testing. Their use was therefore intended to enable a direct comparison of the three material classes rather than to reproduce material-specific certification procedures. The experimental results were subsequently subjected to statistical analysis to assess both within-material relationships and differences between the investigated material classes.

The correlation analyses presented in Section 3.5 were performed using the results obtained for individual specimens. Pearson’s correlation coefficients were calculated to evaluate the relationships between bulk density and the selected mechanical properties. Differences between the investigated materials were additionally evaluated using Welch’s one-way ANOVA, followed by the Games–Howell post-hoc test for pairwise comparisons. This approach was adopted because the groups differed in sample size and variance. A significance level of *p* < 0.05 was used.

The number of specimens used for each laboratory test is summarized in Table 1. All specimens were cylindrical and had a height-to-diameter ratio of approximately 2:1.

#### 2.2.1. Physical Properties

The particle density of the investigated materials was determined using the pycnometer method with water and kerosene (Ekopur U7020), using a procedure based on PN-EN ISO 17892-3:2016-03 [25]. Five independent measurements were performed for each material. Prior to testing, the specimens were dried, ground, and sieved to a particle size below 0.063 mm. Measurements were carried out using 50 mL pycnometers. The masses of both the specimens and the pycnometers were determined with an accuracy of 0.001 g.

Bulk density was determined following a procedure based on PN-EN ISO 17892-2:2015-02 [21] from the measured specimen dimensions and mass. Specimen dimensions were measured with an accuracy of 0.01 mm, while mass was determined using a laboratory balance with an accuracy of 0.01 g.

As the investigated materials exhibited negligible moisture content, the measured bulk density was assumed to be equal to the bulk density of the dry solid skeleton. The porosity of each material was subsequently calculated from the measured particle and bulk densities. Therefore, porosity and bulk density are mathematically dependent variables in the present study and were not treated as fully independent influencing factors in the subsequent analysis.

#### 2.2.2. Uniaxial Compressive Strength Test and Young’s Modulus

The unconfined compressive strength (UCS) was determined following a procedure based on PN-EN ISO 17892-7:2018-05 [22] with a VJTech TRI-SCAN 50 triaxial testing system (VJTech Ltd., Reading, UK) equipped with an automatic force and displacement acquisition system. Axial deformation was determined from the displacement of the loading piston recorded by the testing system, and axial strain was calculated as the recorded piston displacement divided by the initial specimen height.

The tests were performed at an axial strain rate of 0.5–1.5%/min, resulting in a test duration of approximately 5–15 min. Tests were terminated after distinct specimen failure or, if no clear failure occurred, at an axial strain of ε = 15%. For all investigated materials, the reported compressive strength was taken as the maximum stress attained within an axial strain range of up to 10%. No correction for the increase in specimen cross-sectional area resulting from axial deformation was applied. The recorded piston displacement used to determine axial deformation was also used directly, without correction.

Young’s modulus was determined from the approximately linear pre-peak portion of the axial stress–strain curve obtained during unconfined compression under free lateral deformation. The interval was selected individually for each specimen based on curve linearity and the stress level relative to UCS. For Ytong, it generally extended from approximately 10–20% to 75–85% of UCS; for Durafoam, from 10–15% to 70–75%; and for Ekopur U7020, from 10–15% to 80–85%. A linear least-squares regression was applied within the selected interval, and its slope was taken as Young’s modulus.

#### 2.2.3. Triaxial Compressive Strength Tests

**Figure 4:**
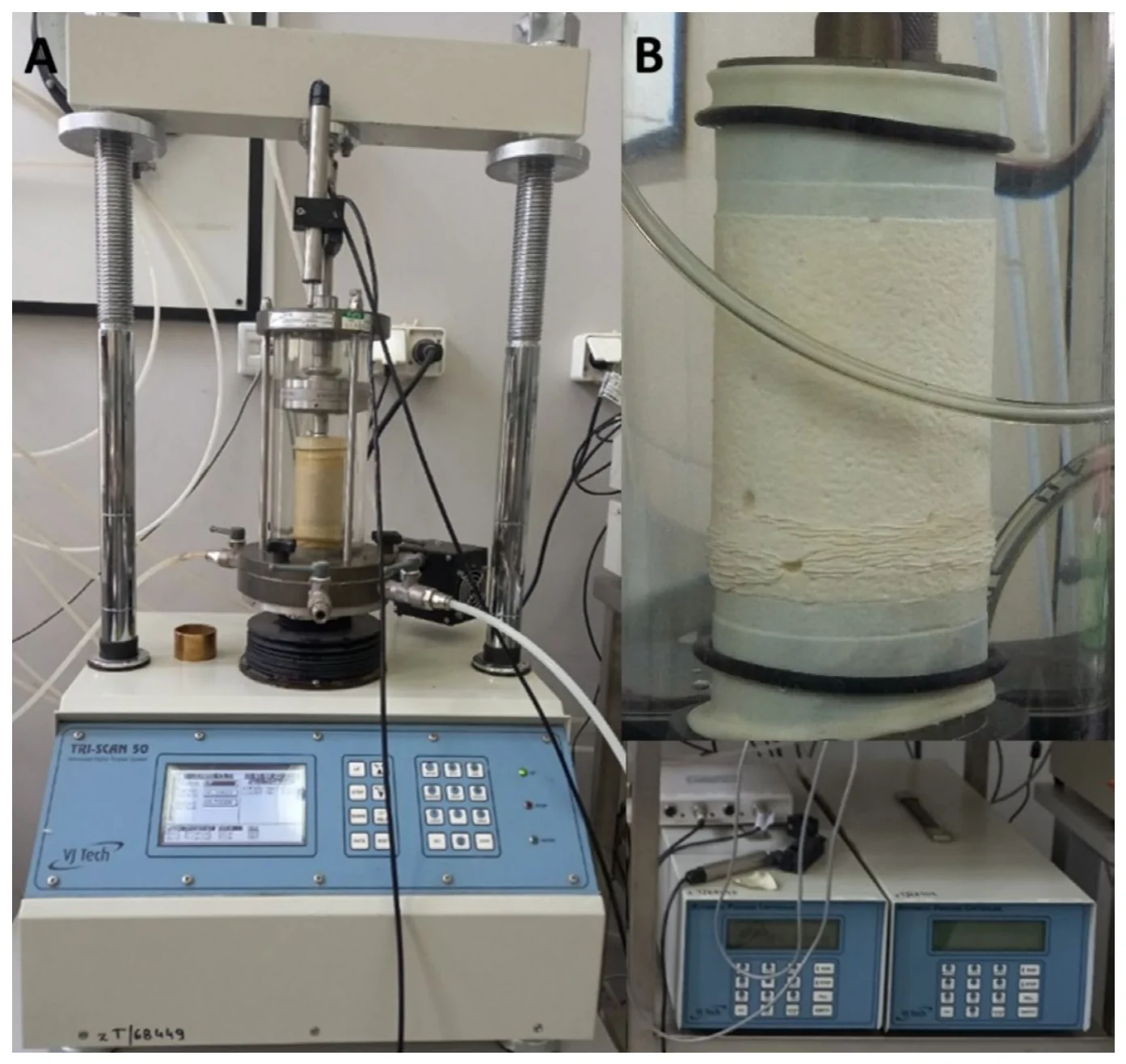
VJTech TRI-SCAN 50 triaxial testing system: (**A**) general view of the apparatus; (**B**) specimen mounted inside the triaxial cell before testing.

Triaxial compression tests were performed following a procedure based on PN-EN ISO 17892-8:2018-05 [23] with a VJTech TRI-SCAN 50 testing system (VJTech Ltd., Reading, UK) (Figure 4). Specimens of foamed concrete and autoclaved aerated concrete measured Ø50 × 100 mm, whereas polyurethane foam specimens measured Ø38 × 76 mm.

The tests were carried out using the unconsolidated undrained (UU) procedure. In the present study, the UU procedure was used primarily as a geomechanical loading framework without a consolidation stage. Because the investigated materials were essentially dry porous solids, the conventional soil-mechanics interpretation of undrained conditions is not directly applicable. The specimens were tested under different confining pressures (σ_3_): 400–2000 kPa for Ytong, 50–600 kPa for Durafoam, and 25–200 kPa for polyurethane foam, while maintaining a constant axial displacement rate of 0.2 mm/min. The confining-pressure ranges were selected according to the substantially different strength levels of the investigated materials. Applying the confinement range used for Ytong to Durafoam or Ekopur U7020 would have caused excessive pre-compression or damage before axial loading. Therefore, lower confinement levels were adopted for Durafoam and Ekopur U7020, while several pressure levels were maintained for each material to determine its failure envelope. Accordingly, the derived shear strength parameters correspond to the material-specific confining-pressure ranges used to establish the respective failure envelopes.

The failure point was defined as the maximum deviator stress. For materials that did not exhibit a distinct peak, failure was assumed to occur at an axial strain of ε = 15%.

The shear strength parameters were determined using the *p*–*q* stress path, defined as

(1) $$p=\frac{\left(\sigma_{1}+\sigma_{3}\right)}{2},$$

and

(2) $$q=\frac{\left(\sigma_{1}-\sigma_{3}\right)}{2},$$

where σ_1_ is the major principal stress, σ_3_ is the confining pressure, and *p* and *q* are the coordinates of the adopted stress space.

The failure points were fitted using the least-squares method according to

*q* = *c_Tu_* + *ptanφ_Tu_*, (3)

where *c_Tu_* and *φ_Tu_* denote the modified shear strength parameters for total stresses.

The Mohr–Coulomb shear strength parameters were then calculated from

*φ_u_* = arcsin(*tanφ_Tu_*), (4)

and

(5) $$c_{u}=\frac{c_{\mathrm{Tu}}}{{\mathrm{cos\varphi}}_{u}},$$

where *φᵤ* is the internal friction angle and *cᵤ* is the cohesion determined for total stresses.

## 3. Results

### 3.1. Particle Density, Bulk Density and Porosity

The highest mean particle density was recorded for Durafoam foamed concrete (2278 ± 11 kg/m^3^) (Table 2), whereas the lowest value was obtained for Ekopur U7020 polyurethane foam (1168 ± 12 kg/m^3^). The particle density of Ytong autoclaved aerated concrete was 2070 ± 22 kg/m^3^. A relatively small scatter of the results was observed for all investigated materials, indicating good repeatability of the pycnometer measurements.

The mean bulk density of the investigated materials ranged from 82 ± 3 kg/m^3^ for Ekopur U7020 polyurethane foam to 566 ± 10 kg/m^3^ for Ytong autoclaved aerated concrete (Table 3). Durafoam foamed concrete exhibited an intermediate bulk density of 518 ± 42 kg/m^3^.

**Figure 5:**
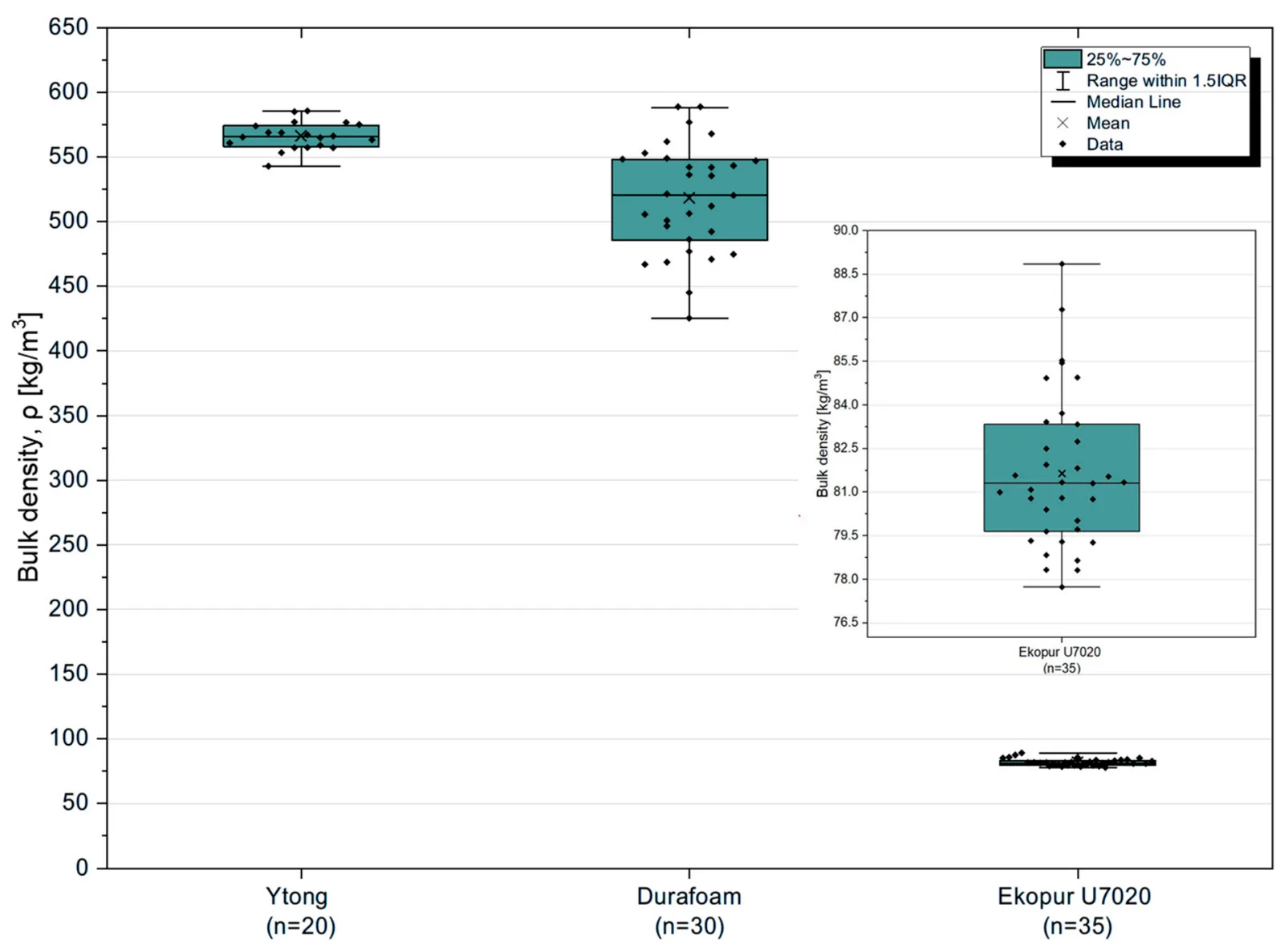
Distribution of bulk density values for the studied materials. The inset shows an enlarged view of the bulk density distribution for Ekopur U7020.

The highest variability in bulk density was observed for Durafoam foamed concrete (CV = 8.02%), whereas the coefficients of variation for Ytong and Ekopur U7020 were 1.83% and 3.16%, respectively. This indicates greater material heterogeneity compared with the other investigated materials, which is also reflected in the distribution of bulk density values shown in Figure 5.

**Figure 6:**
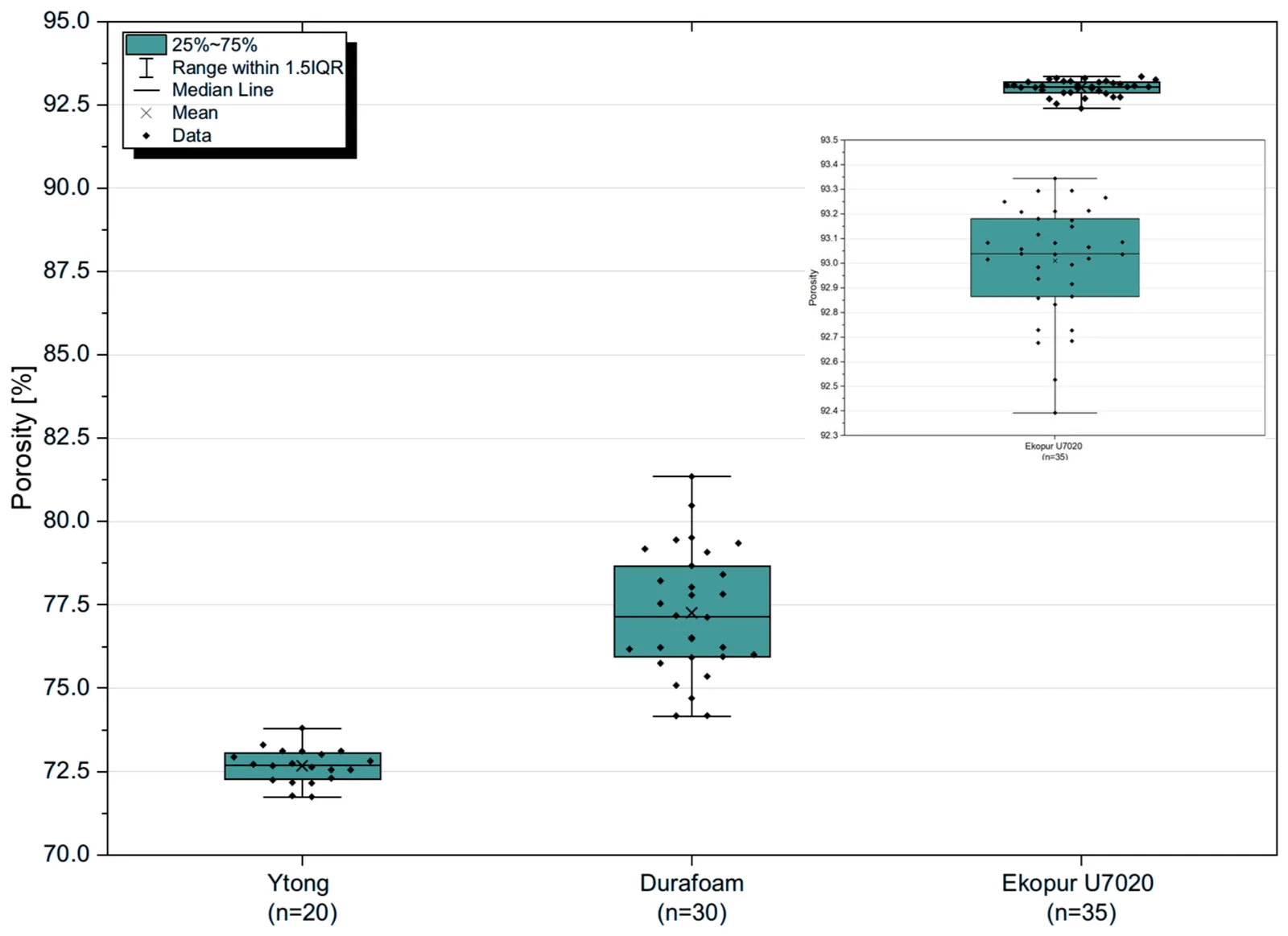
Distribution of porosity values for the studied materials. The inset shows an enlarged view of the porosity distribution for Ekopur U7020.

The highest mean porosity was recorded for Ekopur U7020 polyurethane foam (93 ± 0%), followed by Durafoam foamed concrete (77 ± 2%), whereas the lowest porosity was observed for Ytong autoclaved aerated concrete (73 ± 1%) (Table 4). As in the case of bulk density, the greatest scatter in porosity values was observed for Durafoam foamed concrete (CV = 2.36%), whereas Ytong and Ekopur U7020 exhibited considerably lower variability (CV = 0.69% and 0.24%, respectively), as reflected in the distribution of results presented in Figure 6.

The observed differences in bulk density and porosity served as the basis for further analysis of their influence on the unconfined compressive strength and shear strength parameters of the investigated materials.

### 3.2. Unconfined Compressive Strength and Young’s Modulus

**Figure 7:**
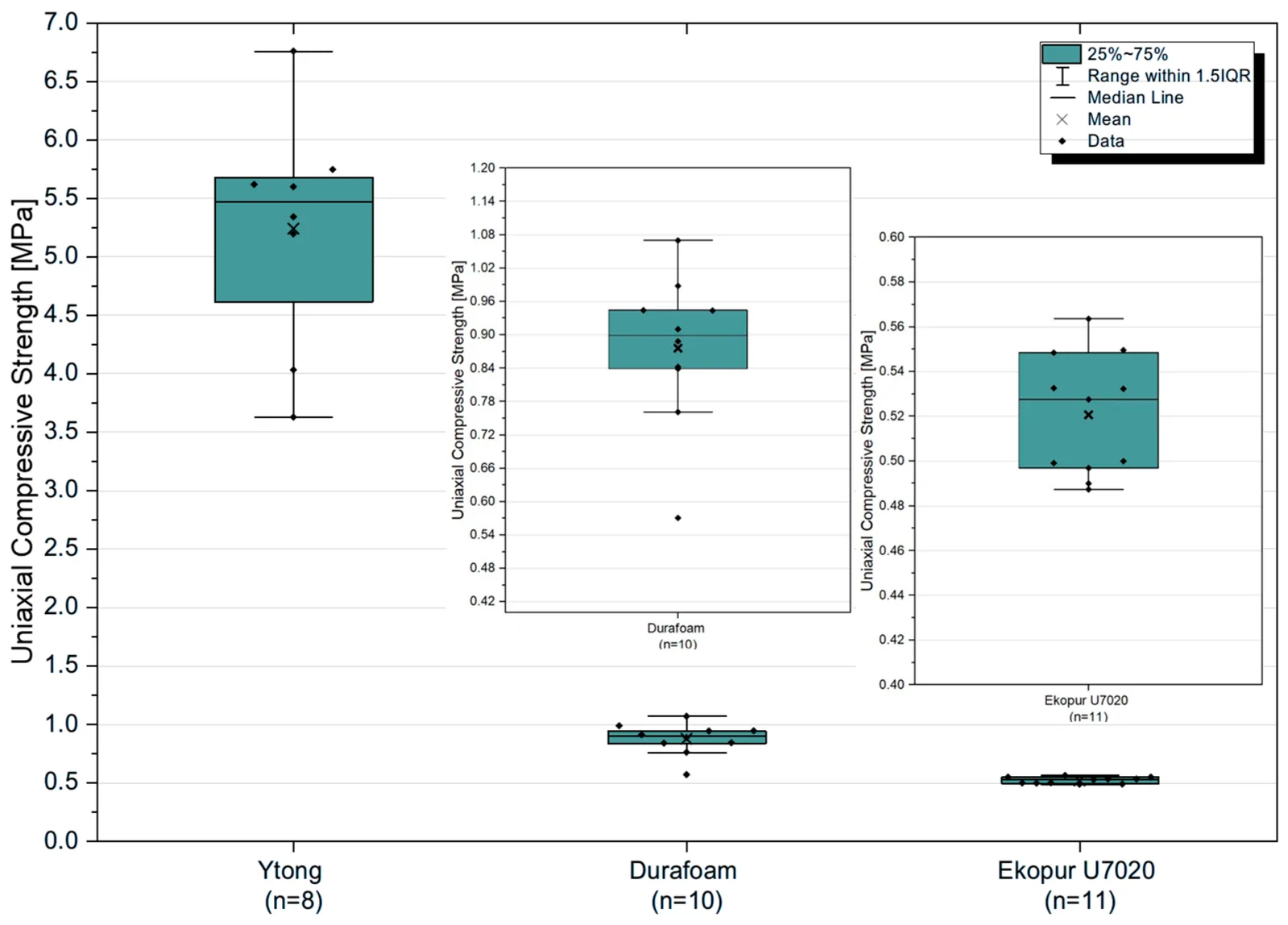
Distribution of unconfined compressive strength (UCS) values for the studied materials. The insets show enlarged views of the UCS distributions for Durafoam and Ekopur U7020.

The mean values of unconfined compressive strength (UCS) are presented in Table 5, while the distribution of the results is shown in Figure 7. The highest mean UCS was obtained for Ytong autoclaved aerated concrete (5.24 ± 0.99 MPa) (Table 5), whereas the lowest value was recorded for Ekopur U7020 polyurethane foam (0.52 ± 0.03 MPa) (Table 5). Durafoam foamed concrete exhibited an intermediate UCS value of 0.88 ± 0.14 MPa (Table 5).

The greatest variability in UCS was observed for the Ytong specimens (CV = 18.95%), whereas the lowest variability was recorded for Ekopur U7020 polyurethane foam (CV = 4.94%). The distribution of the results shown in Figure 7 confirms the greater scatter of the UCS values for Ytong and Durafoam compared with the polyurethane foam.

**Figure 8:**
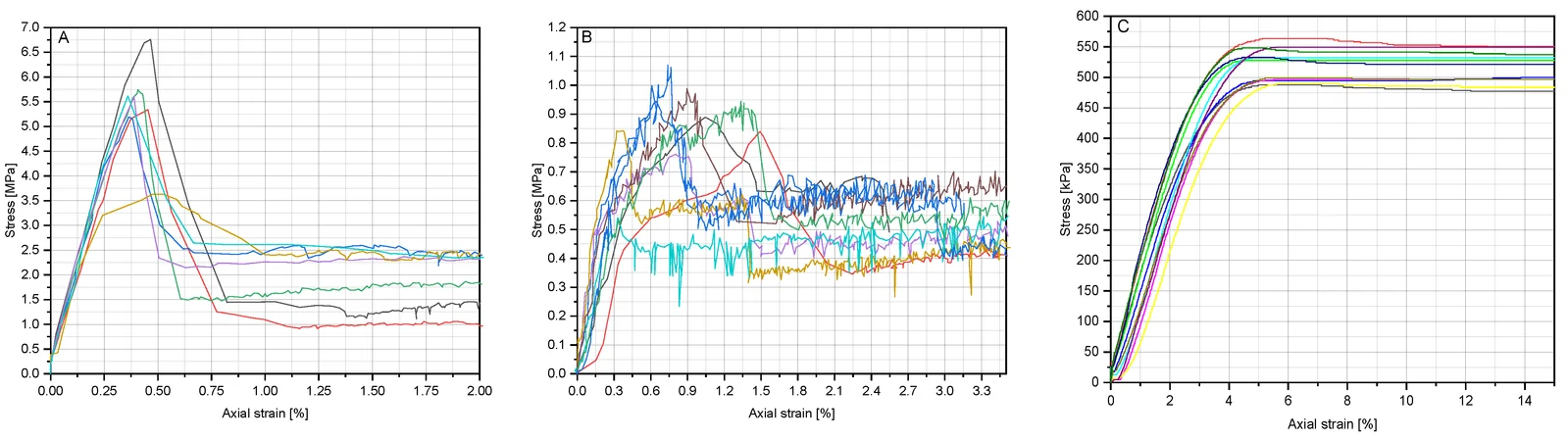
Stress–strain curves of the studied materials: (**A**) Ytong; (**B**) Durafoam; (**C**) Ekopur U7020.

Representative stress–strain curves are presented in Figure 8. Ytong autoclaved aerated concrete and Durafoam foamed concrete exhibited similar stress–strain responses, characterized by a rapid increase in stress up to the peak value, followed by a sudden stress drop. In the case of Ytong, the peak stress was reached at an axial strain of approximately 0.4%, whereas for Durafoam it occurred within the range of approximately 0.4–1.5%. A different behavior was observed for Ekopur U7020 polyurethane foam. The maximum stress was reached at an axial strain of approximately 4–5%, after which the stress remained nearly constant without distinct brittle failure. This stress–strain response indicates a considerably greater deformation capacity compared with the cement-based materials.

The highest Young’s modulus was obtained for Ytong autoclaved aerated concrete (1439.94 ± 129.09 MPa), whereas the lowest value was recorded for Ekopur U7020 polyurethane foam (14.81 ± 1.21 MPa) (Table 6). Durafoam foamed concrete exhibited an intermediate value of 172.30 ± 37.26 MPa (Table 6). Similar to the UCS results, the greatest variability in Young’s modulus was observed for Durafoam foamed concrete (CV = 21.63%) (Table 6).

**Figure 9:**
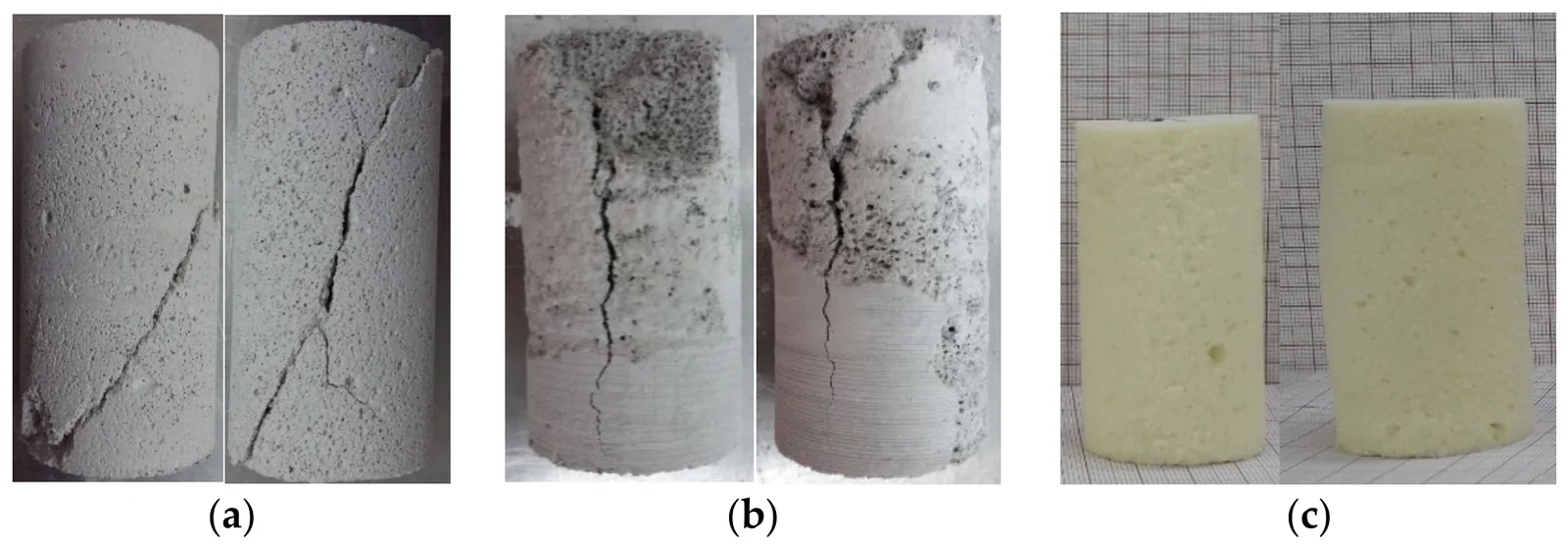
Representative specimens after the unconfined compression test: (**a**) Ytong; (**b**) Durafoam; (**c**) Ekopur U7020.

Representative failure patterns after the unconfined compression tests are presented in Figure 9. Both autoclaved aerated concrete and foamed concrete failed through the development of longitudinal cracks, leading to brittle specimen fragmentation. In contrast, no distinct cracking or brittle failure was observed for the polyurethane foam. Instead, the dominant failure mechanism was substantial material deformation associated with its cellular structure.

### 3.3. Cohesion and Internal Friction Angle

**Figure 10:**
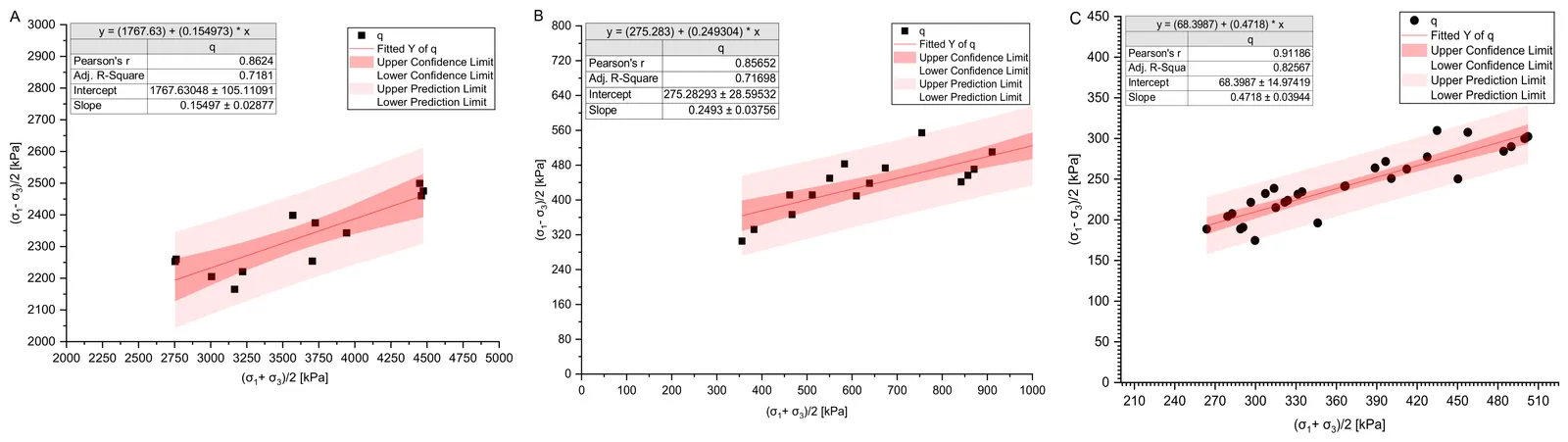
Stress paths in the *p*–*q* plane: (**A**) Ytong; (**B**) Durafoam; (**C**) Ekopur U7020.

The shear strength parameters were determined based on the analysis of the p–q stress paths. The failure points together with the regression lines obtained using the least-squares method are presented in Figure 10. The resulting values of cohesion and internal friction angle are summarized in Table 7.

The highest cohesion was obtained for Ytong autoclaved aerated concrete, reaching 1.79 MPa. The cohesion of Durafoam foamed concrete was approximately six times lower (0.284 MPa), whereas the lowest value was obtained for Ekopur U7020 polyurethane foam (0.078 MPa). An opposite trend was observed for the internal friction angle. The lowest internal friction angle was recorded for Ytong (8.92°), followed by Durafoam foamed concrete (14.44°), whereas the highest value was obtained for Ekopur U7020 polyurethane foam (28.15°).

For all investigated materials, the failure points exhibited a linear relationship in the *p*–*q* stress space, which enabled the determination of the Mohr–Coulomb shear strength parameters by linear regression. The coefficient of determination (R^2^) ranged from 0.7170 for Durafoam to 0.8257 for Ekopur U7020 polyurethane foam, indicating a good fit of the linear model to the experimental data (Figure 10) despite some scatter of the data points in the p–q space.

**Figure 11:**
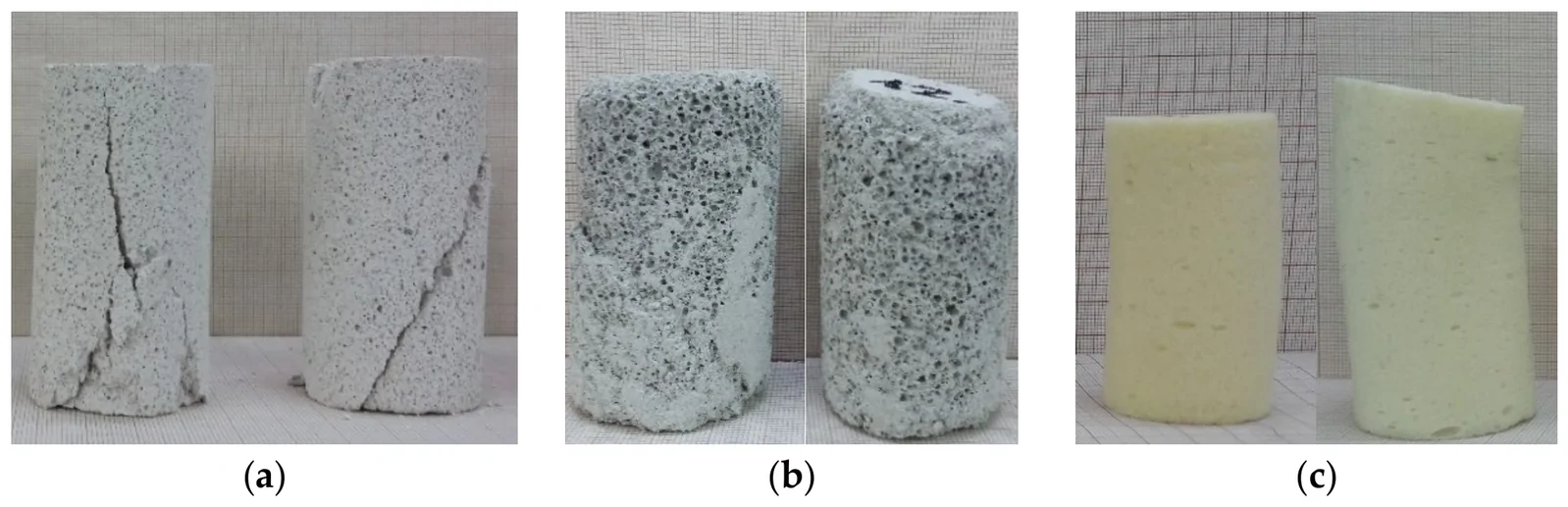
Representative specimens after the triaxial compression test: (**a**) Ytong; (**b**) Durafoam; (**c**) Ekopur U7020.

Representative specimens after the triaxial compression tests are shown in Figure 11. Both autoclaved aerated concrete and foamed concrete exhibited distinct cracking accompanied by localized material failure. In contrast, the polyurethane foam underwent substantial deformation without the characteristic brittle fracture observed in the cement-based materials.

### 3.4. Comparative Summary of the Investigated Materials

To provide a comprehensive comparison of the investigated materials, all determined physical and mechanical properties are summarized in Table 8.

Welch’s ANOVA revealed significant differences among the investigated materials in bulk density [F(2, 31.94) = 21,028.05, *p* < 0.0001], UCS [F(2, 10.82) = 115.03, *p* < 0.0001], and Young’s modulus [F(2, 8.01) = 451.35, *p* < 0.0001]. Games–Howell post-hoc comparisons showed that all pairwise differences between Ytong, Durafoam, and Ekopur U7020 were statistically significant for each of these properties (*p* < 0.001).

Comparison of the results revealed substantial differences in the mechanical properties of the investigated materials. Ytong autoclaved aerated concrete exhibited the highest unconfined compressive strength, the highest Young’s modulus, and the greatest cohesion.

Particular attention should be paid to the comparison between Ytong and Durafoam. Despite their very similar bulk densities (566 and 518 kg/m^3^, respectively), the UCS of Ytong was approximately six times higher, while its Young’s modulus was more than eight times greater than that of Durafoam. A similar relationship was observed for the cohesion determined from the triaxial compression tests.

At the same time, Durafoam exhibited the highest particle density among all investigated materials; however, this did not translate into the highest mechanical properties.

Ekopur U7020 exhibited the lowest bulk density and the highest porosity. At the same time, it showed the lowest compressive strength, Young’s modulus, and cohesion, while exhibiting the highest internal friction angle.

**Figure 12:**
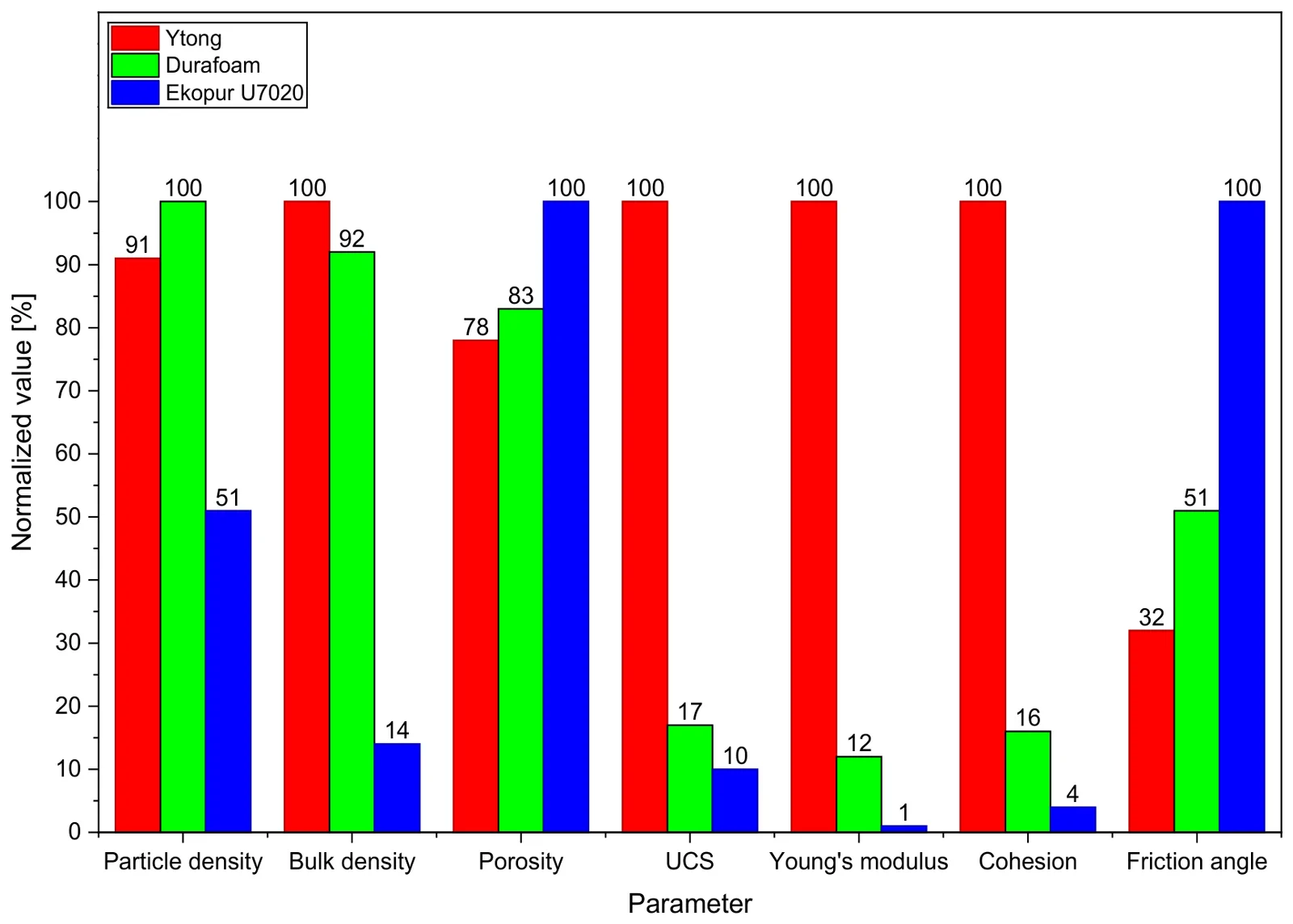
Comparison of the normalized physical and mechanical properties of the studied materials. The maximum value of each parameter was assumed to be 100%.

To facilitate a direct comparison of the physical and mechanical properties of the investigated materials, normalized values of all analyzed parameters are presented in Figure 12. For each parameter, the highest measured value was taken as 100%, while the remaining values were expressed as percentages of this reference value.

Figure 12 clearly demonstrates that autoclaved aerated concrete and foamed concrete exhibit similar bulk densities but markedly different mechanical properties, particularly with respect to compressive strength, Young’s modulus, and cohesion. This confirms that similar bulk density does not necessarily imply similar mechanical behavior for materials belonging to different material classes.

Overall, the comparison indicates that the investigated materials exhibit distinctly different mechanical characteristics despite partially similar physical properties. This is particularly evident for autoclaved aerated concrete and foamed concrete, whose bulk densities differed by only approximately 48 kg/m^3^, whereas the compressive strength, Young’s modulus, and cohesion of autoclaved aerated concrete were approximately six, eight, and six times higher, respectively.

### 3.5. Influence of Bulk Density on Mechanical Properties

To evaluate the influence of bulk density variability on the mechanical properties, the relationships between bulk density and unconfined compressive strength, as well as between bulk density and Young’s modulus, were analyzed using the results obtained for individual specimens. The analyses were performed separately for each material, allowing the influence of small variations in bulk density within the same material group to be assessed.

**Figure 13:**
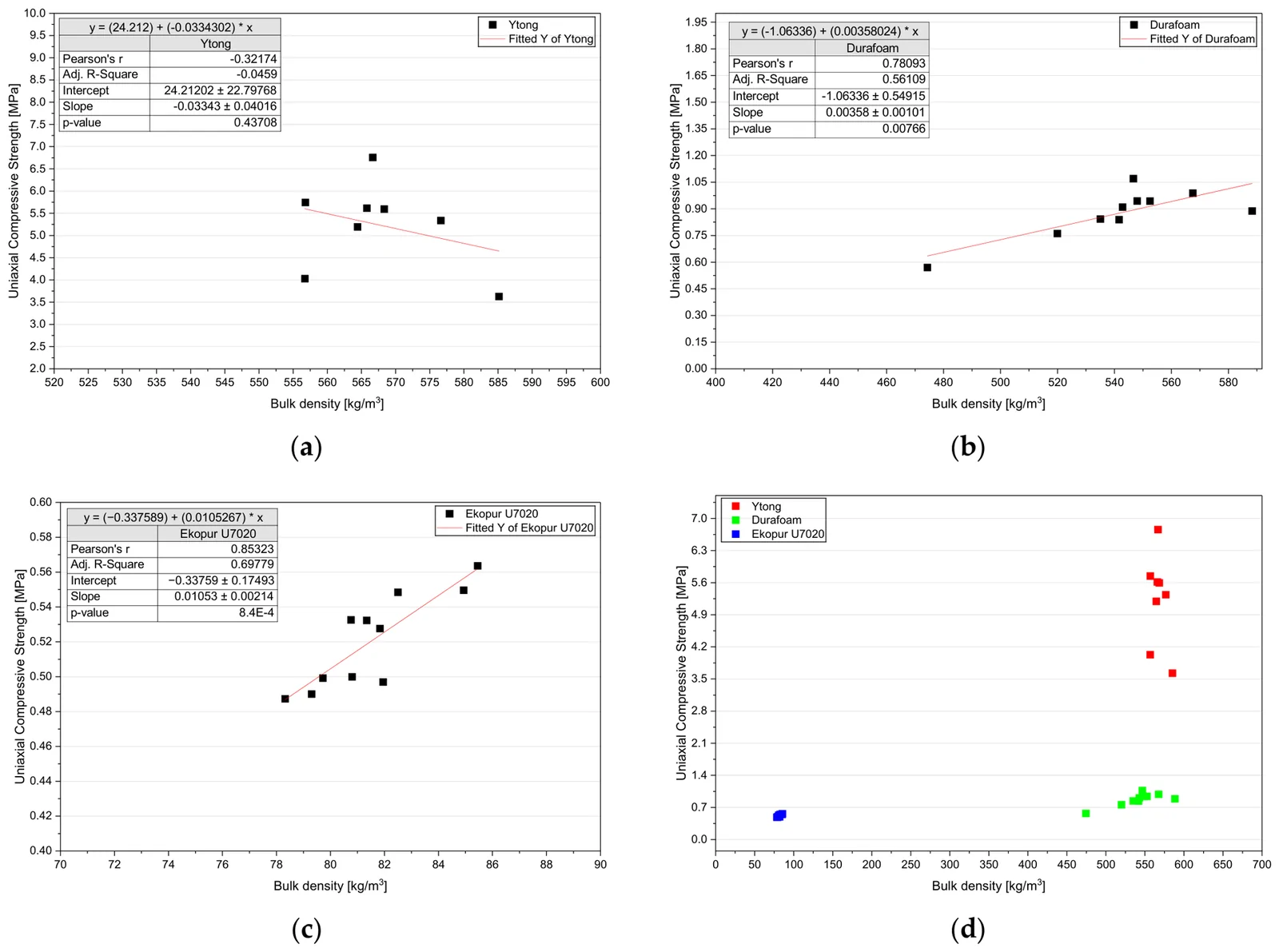
Relationship between bulk density and unconfined compressive strength (UCS) for: (**a**) Ytong; (**b**) Durafoam; (**c**) Ekopur U7020; and (**d**) all tested specimens.

The relationships between bulk density and unconfined compressive strength are presented in Figure 13.

The results showed that the influence of bulk density on compressive strength was not the same for all investigated materials. For Durafoam and Ekopur U7020, a positive relationship between bulk density and UCS was observed. The correlation was strong and statistically significant for both Durafoam (*r* = 0.78, *p* = 0.008) and Ekopur U7020 (*r* = 0.85, *p* < 0.001).

For Ytong, the correlation was weak and not statistically significant (*r* = −0.32, *p* = 0.437).

These results indicate that the influence of bulk density on compressive strength depends on the type of material and cannot be described by a single universal relationship applicable to all lightweight porous materials.

**Figure 14:**
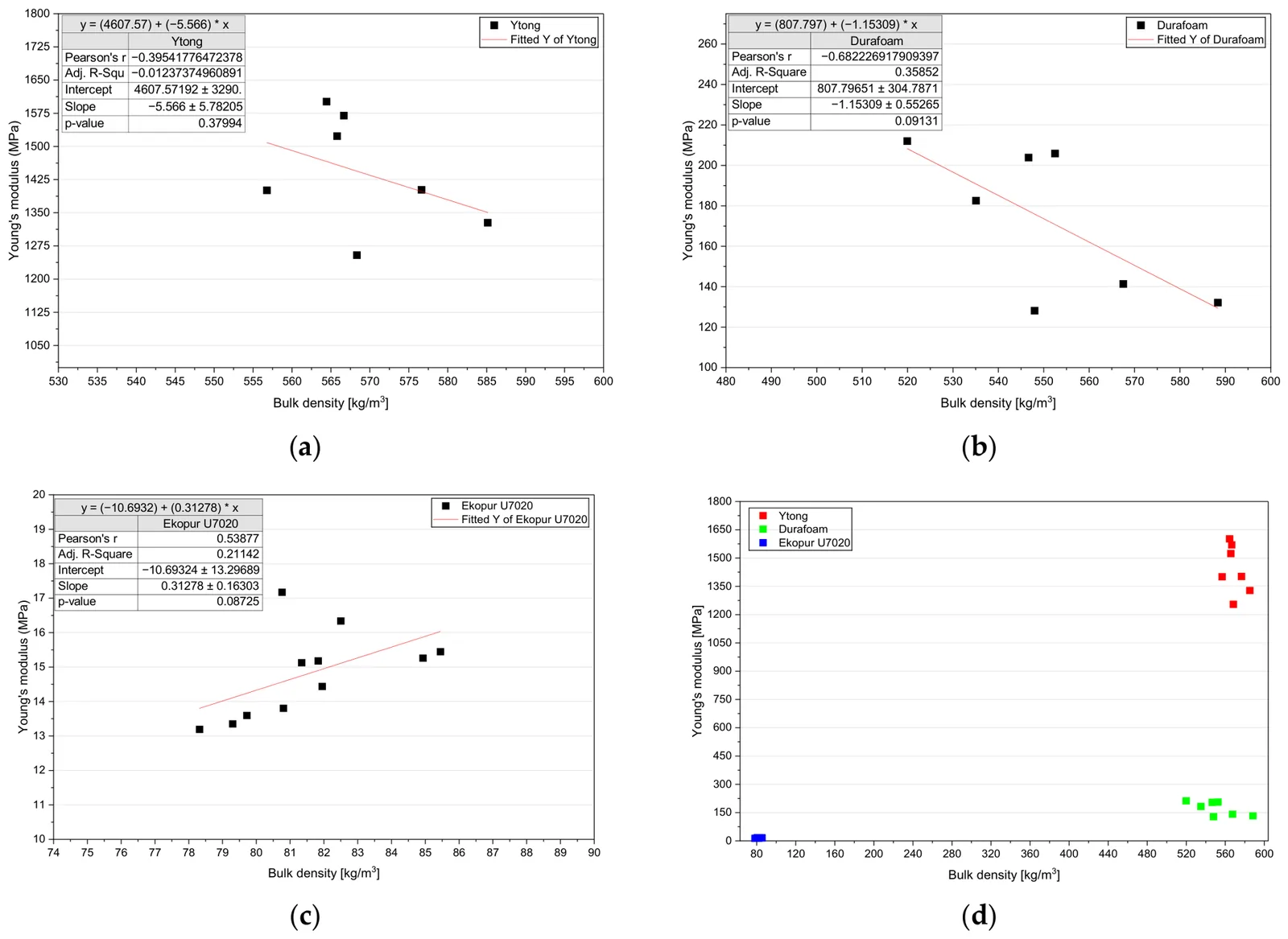
Relationship between bulk density and Young’s modulus for: (**a**) Ytong; (**b**) Durafoam; (**c**) Ekopur U7020; and (**d**) all tested specimens.

An analogous analysis was performed for the relationship between bulk density and Young’s modulus (Figure 14).

For Ekopur U7020, a moderate positive correlation was observed between bulk density and Young’s modulus (*r* = 0.54, *p* = 0.087). In contrast, Durafoam exhibited a moderate negative correlation (*r* = −0.68, *p* = 0.091), while Ytong showed a weak negative correlation (*r* = −0.40, *p* = 0.380). None of these correlations was statistically significant at the 0.05 significance level. Therefore, the observed differences in the direction and magnitude of the correlation coefficients should be interpreted with caution.

Because porosity plays an important role in determining the properties of lightweight porous materials, the relationships between porosity and both unconfined compressive strength and Young’s modulus were also analyzed. Since porosity was calculated from bulk density and particle density, the resulting relationships exhibited the same characteristics but with opposite signs of the correlation coefficients. Consequently, increasing porosity corresponded to decreasing bulk density and led to the same conclusions regarding the influence of physical parameters on the mechanical properties of the investigated materials. Therefore, the corresponding plots for porosity are not presented, as they do not provide additional information beyond that obtained from the bulk density analysis.

A comparison of all analyzed relationships demonstrated that bulk density was not related to the mechanical properties in the same manner for all investigated materials. For Durafoam and Ekopur U7020, statistically significant positive correlations were obtained between bulk density and UCS, whereas the correlation for Ytong was not statistically significant. For Young’s modulus, none of the within-material correlations was statistically significant, despite differences in the direction and magnitude of the Pearson coefficients. These results indicate that the observed density–property relationships differ among the investigated materials; however, the within-material correlations should be interpreted cautiously because of the limited sample sizes and relatively narrow ranges of bulk density.

## 4. Discussion

The key finding of the present study is that the results do not support a universal relationship between bulk density and the mechanical properties of the investigated materials. This is particularly evident when comparing autoclaved aerated concrete and Durafoam foamed concrete. Despite their similar bulk densities (566 and 518 kg/m^3^, respectively), these materials exhibited approximately a sixfold difference in unconfined compressive strength, an eightfold difference in Young’s modulus, and a sixfold difference in cohesion. These results indicate that similar bulk density does not necessarily imply similar mechanical behavior. Consequently, bulk density alone is not a sufficient parameter for predicting the mechanical properties of materials belonging to different classes of lightweight porous materials.

The observed differences may be associated with differences in microstructural characteristics, including pore size, shape, distribution, and pore connectivity, as well as with the properties of the solid skeleton. However, since direct microstructural characterization was not performed in the present study, these factors should be regarded as literature-supported explanations rather than mechanisms demonstrated experimentally herein. As emphasized by Qu and Zhao [9], the properties of autoclaved aerated concrete are governed not only by total porosity but also by pore size, shape, distribution, pore connectivity, and the properties of the solid phase. Similar observations were reported by Yu et al. [14] and Omran et al. [15], who demonstrated that, even at the same density, variations in microstructure lead to significant differences in the mechanical properties of cellular materials. The importance of microstructure was also confirmed by Roobankumar et al. [24], who showed that the mechanical properties of lightweight concrete containing waste polyurethane foam aggregate are closely related to its internal structure. Their SEM analysis demonstrated that the quality of the interfacial transition zone between the polyurethane foam aggregate and the cement matrix has a significant influence on the resulting strength parameters.

The present results are also consistent with the observations of Chung et al. [7], who compared lightweight cementitious materials with similar bulk densities and demonstrated that their mechanical properties may differ considerably because of differences in microstructure. In their study, lightweight aggregate concrete and foamed concrete were compared at target density levels of 500, 750, and 1000 kg/m^3^, and the compressive strength of the lightweight aggregate concrete was more than twice that of the corresponding foamed concrete at each density level. Similar conclusions were reported by Roobankumar and SenthilPandian [16] for polyurethane foam concrete, showing that changes in material composition affect both bulk density and mechanical properties, while the relationship between these parameters is not linear. After 28 days, the compressive strength decreased from 30.5 MPa for the control concrete to 17.4 MPa at 60% polyurethane foam aggregate replacement, corresponding to a reduction of approximately 43%. Likewise, Harith [26] demonstrated that the mechanical properties of polyurethane foamed concrete depend not only on density but also on curing conditions and the internal structure of the material. At very similar hardened-concrete densities of 1640 and 1650 kg/m^3^, the 28-day compressive strength ranged from 11.1 to 29.2 MPa for the mixture with fly ash and from 10.3 to 24.2 MPa for the mixture without fly ash, depending on the curing regime.

Unlike most previous studies, which have examined density–property relationships within individual material classes, the present study directly compares three fundamentally different classes of lightweight porous materials within a common experimental and analytical framework. The scientific insight obtained from this cross-class comparison is not merely that density alone is insufficient to describe mechanical behavior, which is already recognized in the literature, but that the magnitude and character of density–property relationships differ substantially between material classes. The comparison between AAC and Durafoam presented above provides a clear example of this cross-class difference. Thus, density–property relationships established for one class of lightweight porous materials cannot be assumed to be directly transferable to another class.

These findings are consistent with conclusions presented in recent review papers. Liu et al. [4], Ortiz-Marqués et al. [17], and Peng et al. [8] emphasized that a reliable assessment of lightweight porous materials requires the combined analysis of physical, microstructural, and mechanical parameters. Fu et al. [19] and Sifan et al. [18] similarly highlighted the limitations of relying on a single parameter, such as bulk density, to predict the behavior of lightweight materials.

The common experimental and analytical framework enabled the cross-class differences to be quantified using the same set of physical and mechanical parameters, providing a comparative dataset that reveals substantial differences in UCS, Young’s modulus, cohesion, and internal friction angle between material classes of similar bulk density.

The correlation analysis demonstrated that the relationship between bulk density and mechanical properties depends on the type of material. The obtained correlation coefficients differed among the investigated materials, suggesting that factors other than bulk density contribute to the observed mechanical response. These may include the microstructural characteristics discussed above. Because porosity was calculated from particle density and bulk density, porosity and bulk density are not statistically independent variables in the present study. Moreover, the obtained results indicate that neither parameter alone provides a universal basis for predicting the mechanical properties of different classes of lightweight porous materials.

The obtained results are consistent with the observations of Qu and Zhao [9] for autoclaved aerated concrete and Uysal et al. [27] for lightweight aggregate concrete, further supporting the material-specific character of density–property relationships. In the studies summarized by Qu and Zhao [9], AAC with densities of 500–650 kg/m^3^ exhibited compressive strengths of 2.9–3.3 MPa, whereas AAC with densities of 570–620 kg/m^3^ reached 4.3–6.1 MPa, showing that similar density ranges may correspond to different strength levels. Uysal et al. [27] reported dry compressive strengths of 18–38 MPa for LECA concrete, while the 28-day static Young’s modulus ranged from 8.8 to 19.6 GPa in the dry state and from 12.3 to 24.5 GPa in the moist state. Similar conclusions were reported by Shivakumar and Deb [20] for rigid polyurethane foams, whose mechanical properties were shown to depend not only on density but also on the geometry and morphology of the cellular structure.

From a practical perspective, these findings indicate that bulk density should primarily be regarded as a classification and technological parameter rather than as a universal criterion for assessing the strength and stiffness of different classes of lightweight porous materials. The results further suggest that density–property relationships should be modeled separately for individual material groups. Consequently, the selection of lightweight porous materials for engineering applications should be based on a comprehensive physical and mechanical characterization that considers both microstructural characteristics and the mechanical response of the material under loading.

The within-material correlation results should be interpreted with caution because of the limited sample sizes and relatively narrow ranges of bulk density. Although statistically significant positive correlations between bulk density and UCS were obtained for Durafoam and Ekopur U7020, the corresponding correlation for Ytong was not statistically significant. Moreover, none of the correlations between bulk density and Young’s modulus reached statistical significance at the 0.05 level. Therefore, the observed positive or negative trends, particularly for Young’s modulus, should not be interpreted as conclusive evidence of density-dependent mechanical behavior. In addition, Young’s modulus was determined from axial strain based on loading-piston displacement; therefore, the reported values should be interpreted within the adopted testing configuration.

Another important practical outcome of this study is the potential application of the obtained physical and mechanical dataset in the numerical analysis of lightweight porous materials. The determined values of particle density, bulk density, porosity, Young’s modulus, unconfined compressive strength, and shear strength parameters obtained from triaxial compression tests constitute a consistent set of material properties that may serve as a reference for the calibration and evaluation of constitutive models used in finite element analyses. The dataset may also support future comparative numerical studies of the investigated material classes.

It should be emphasized, however, that the present study included three representative materials belonging to different classes of lightweight porous materials. Therefore, the obtained results apply specifically to the investigated materials and should not be directly generalized to all types of autoclaved aerated concrete, foamed concrete, or rigid polyurethane foams. The resulting comparative dataset may nevertheless serve as a basis for future investigations involving a wider range of densities, material compositions, and manufacturing technologies.

A limitation of the present study is the absence of direct microstructural characterization; therefore, the interpretation of the role of pore structure and solid-skeleton characteristics is based on previous literature rather than direct experimental evidence. Future research will include X-ray micro-computed tomography (micro-CT) and scanning electron microscopy (SEM), following methodological approaches applied in recent studies of cement-based materials [28,29]. Such analyses would enable direct quantitative relationships between microstructural parameters and the mechanical properties of lightweight porous materials to be established.

A methodological limitation of the present study is that the PN-EN ISO 17892 series was applied to materials outside its primary geotechnical scope. Nevertheless, the adopted procedures are based on standard density measurements and established unconfined and triaxial compression principles, providing a consistent basis for comparison between the investigated materials. The main limitation therefore concerns the formal scope and interpretation of the procedures rather than the comparability of the experimental results within the present study.

## 5. Conclusions

This study presented a comparative physical and mechanical characterization of three lightweight porous materials representing different material classes: autoclaved aerated concrete, foamed concrete, and rigid polyurethane foam. A common experimental and analytical framework enabled their direct comparison using the same set of physical and mechanical parameters.

The results showed that similar bulk density does not necessarily correspond to similar mechanical properties. This was particularly evident from the comparison between autoclaved aerated concrete and foamed concrete, which, despite having similar bulk densities, exhibited substantially different values of unconfined compressive strength, Young’s modulus, and shear strength parameters determined from triaxial compression tests. These findings indicate that the relationships between bulk density and mechanical properties are material-dependent and should not be assumed to be directly transferable between different classes of lightweight porous materials.

Bulk density alone did not adequately explain the observed differences in mechanical behavior. Based on previous studies, these differences may also be associated with microstructural characteristics and the properties of the solid skeleton; however, these factors were not directly investigated here. Because porosity was calculated from particle density and bulk density, porosity and bulk density should not be interpreted as statistically independent variables.

The results support the importance of a combined physical and mechanical characterization of lightweight porous materials rather than evaluation based on a single physical parameter.

The principal contribution of the present study is the quantitative cross-class comparison of three lightweight porous materials with fundamentally different matrices and pore-formation mechanisms that use a common experimental and analytical framework. The results demonstrate that density–property relationships derived for one material class should not be assumed to be directly transferable to another. In particular, similar bulk density may coexist with several-fold differences in unconfined compressive strength, Young’s modulus, and cohesion. This finding provides an experimental basis for treating bulk density primarily as a material-specific classification parameter rather than as a universal predictor of mechanical properties across different classes of lightweight porous materials.

The consistent dataset of physical and mechanical properties obtained in this study may serve as a valuable reference for the calibration and evaluation of numerical models of lightweight porous materials. Because the investigated parameters were determined within a common experimental and analytical framework, the dataset provides a consistent basis for future comparative studies and may support numerical analyses of the investigated material classes.

## Figures and Tables

**Table 1:** Number of specimens used for each laboratory test.

Material	Specimen Dimensions [mm]	Particle Density	Bulk Density and Porosity	UCS	Triaxial Compression Tests
Ytong	Ø50 × 100	5	20	8	12
Durafoam	Ø50 × 100	5	30	10	20
Ekopur U7020	Ø38 × 76	5	35	11	24

**Table 2:** Particle density of the studied materials.

Material	*n* [-]	Min [kg/m^3^]	Max [kg/m^3^]	Mean ± SD [kg/m^3^]
Ytong	5	2043	2106	2070 ± 22
Durafoam	5	2257	2289	2278 ± 11
Ekopur U7020	5	1153	1182	1168 ± 12

*n*—number of specimens.

**Table 3:** Bulk density of the studied materials.

Material	*n* [-]	Min [kg/m^3^]	Max [kg/m^3^]	Mean ± SD [kg/m^3^]	CV [%]
Ytong	20	542	585	566 ± 10	1.83
Durafoam	30	425	588	518 ± 42	8.02
Ekopur U7020	35	78	89	82 ± 3	3.16

SD—standard deviation. CV—coefficient of variation.

**Table 4:** Porosity of the studied materials.

Material	*n* [-]	Min [%]	Max [%]	Mean ± SD [%]	CV [%]
Ytong	20	72	74	73 ± 1	0.69
Durafoam	30	74	81	77 ± 2	2.36
Ekopur U7020	35	92	93	93 ± 0	0.24

**Table 5:** Unconfined compressive strength (UCS) of the studied materials.

Material	*n* [-]	Min [MPa]	Max [MPa]	Mean ± SD [MPa]	CV [%]
Ytong	8	3.63	6.76	5.24 ± 0.99	18.95
Durafoam	10	0.57	1.07	0.88 ± 0.14	15.68
Ekopur U7020	11	0.49	0.56	0.52 ± 0.03	4.94

**Table 6:** Young’s modulus of the studied materials.

Material	*n* [-]	Min [MPa]	Max [MPa]	Mean ± SD [MPa]	CV [%]
Ytong	7	1254.26	1601.51	1439.94 ± 129.09	8.97
Durafoam	7	128.14	212.07	172.30 ± 37.26	21.63
Ekopur U7020	11	13.19	17.17	14.81 ± 1.21	8.18

**Table 7:** Shear strength parameters of the studied materials.

Material	*n* [-]	Equation	Pearson’s r	*p*-Value	R^2^	Cohesion [kPa]	Internal Friction Angle [°]
Ytong	12	q = 0.155p + 1767.63	0.8624	<0.001	0.7181	1789.25	8.92
Durafoam	18	q = 0.249p + 275.28	0.8565	<0.001	0.7170	284.26	14.44
Ekopur U7020	24	q = 0.472p + 68.40	0.9119	<0.001	0.8257	77.56	28.15

**Table 8:** Summary of the physical and mechanical properties of the studied materials.

Parameter	Ytong	Durafoam	Ekopur U7020
Particle density [kg/m^3^]	2070 ± 22	2278 ± 11	1168 ± 12
Bulk density [kg/m^3^]	566 ± 10	518 ± 42	82 ± 3
Porosity [%]	73 ± 1	77 ± 2	93 ± 0
UCS [MPa]	5.24 ± 0.99	0.88 ± 0.14	0.52 ± 0.03
Young’s modulus [MPa]	1439.94 ± 129.09	172.30 ± 37.26	14.81 ± 1.21
Cohesion [MPa]	1.789	0.284	0.078
Internal friction angle [°]	8.92	14.44	28.15

## Data Availability

The original contributions presented in the study are included in the article; further inquiries can be directed to the corresponding author.
